# Inhibition of microRNA-103a inhibits the activation of astrocytes in hippocampus tissues and improves the pathological injury of neurons of epilepsy rats by regulating BDNF

**DOI:** 10.1186/s12935-019-0821-2

**Published:** 2019-04-24

**Authors:** Ping Zheng, He Bin, Wei Chen

**Affiliations:** 1 Department of Neurosurgery, Shanghai Pudong New Area People’s Hospital, No 490, South Chuanhuan Road, Shanghai, 201299 People’s Republic of China; 2 Department of Neurosurgery, First affiliated Hospital of Nanchang University, Nanchang, China

**Keywords:** Epilepsy, MicroRNA-103a, BDNF, Astrocyte, Hippocampal neurons, Inflammatory lesions, GFAP, Transmission electron microscope

## Abstract

**Background:**

The aim of this study is to explore the effect of microRNA-103a (miR-103a) on astrocytes activation and hippocampal neuron injury in epilepsy rats by targeting brain-derived neurotrophic factor (BDNF).

**Methods:**

The epilepsy rat model was induced by intraperitoneal injection of lithium chloride-pilocarpine. Successful modeled rats were intralateroventricularly microinjected with miR-103a inhibitors, inhibitors negative control (NC), siRNA-NC and BDNF-siRNA, respectively. The RT-qPCR and western blot analysis were used to detect the expression of miR-103a, BDNF and glial fibrillary acidic protein (GFAP) in hippocampus tissues of rats. TUNEL staining was used to detect the apoptosis of hippocampal neurons. The RT-PCR and ELISA was used to detect the levels of TNF-α and IL-6 in hippocampal tissues and in serum, respectively.

**Results:**

Increased expression of miR-103a, GFAP, and number of apoptotic neurons, decreased expression of BDNF and number of surviving neurons were found in hippocampus tissues of epilepsy rats. After miR-103a inhibitors interfered with epilepsy rats, there showed decreased expression of miR-103a and GFAP, increased expression of BDNF and decreased number of apoptotic neuron as well as increased number of surviving neurons. Compared with miR-103a inhibitors alone, epilepsy rats treated with BDNF-siRNA combined with miR-103a inhibitors significantly increased expression of GFAP in hippocampal tissues of epilepsy rats, increased number of apoptotic neurons and significantly decreased the number of surviving neurons.

**Conclusion:**

Our study provides evidence that the inhibition of miR-103a can inhibit the activation of astrocytes in hippocampus tissues and improve the pathological injury of neurons of epilepsy rats by regulating BDNF gene.

**Electronic supplementary material:**

The online version of this article (10.1186/s12935-019-0821-2) contains supplementary material, which is available to authorized users.

## Background

Epilepsy, a life-shortening brain disorder, is the most frequently serious neurological condition, which affecting about 1% of the population worldwide [1, 2]. Epilepsy is a diverse disease with more than fifteen different seizure types and thirty epilepsy syndromes, which is related to the substantial comorbidity, such as anxiety, depression, and increased mortality [3, 4]. The most commonly-seen risk factors for seizures are infections and infestations, and both of which are the most important and preventable risk factors for epilepsy around the world, especially in resource-poor settings [5–7]. In high-income countries, the incidence rates of epilepsy appear to be declining, and the validity of epilepsy that is diagnosed from different data sources is different, thus the contemporary population-based studies are needed [8]. Since the early 1990s, the different kinds of new antiepileptic drugs (AEDs) have emerged, but the available evidence demonstrates that no substantial improvement is found in terms of the efficacy and tolerability of drug treatment [9, 10]. In view of this, new concepts along with fresh thinking is needed to radically improve AED development, and this study may help to seek for more effective drug treatment of epilepsy in the near future.

MicroRNAs (miRNAs) consists of a class of small RNAs, which have some important cell functions, including proliferation, differentiation as well as metabolism [11, 12]. Growing evidence has revealed that miRNAs are participated in the pathogenesis of most cancers, which could both function as oncogenes or tumor suppressors [13, 14]. Among which, miR-103 is one of the members of the miR-15/107 family [15]. Several studies have shown that the downregulation of miR-103 may induces various diseases, including diabetes [16], cancer [17] as well as myocardial infarction [18]. Microarray information showed that miR-103 was highly expressed in the central nervous system and further upregulated after stroke [19]. Additionally, evidence has shown that inhibition of miR-103 can up-regulate NCX1 gene, thus reduce the volume of cerebral infarction after stroke, and improve the neurological deficit after stroke [20]. These results suggest that the upregulation of miR-103 may play an important role in the regulation of gene expression in the central nervous system. However, whether miR-103 is involved in the occurrence and the development of epilepsy has never been discussed. Brain-derived neurotrophic factor (BDNF) belongs to the neurotrophin family, and it functions in neurite outgrowth, neural differentiation, synaptic plasticity as well as survival of nerve cells [21]. Besides, BDNF is expressed throughout the mammalian brain, such as the hippocampus, cerebral cortex, basal forebrain, striatum, cerebellum, hypothalamus as well as limbic structures [22]. It is reported that BDNF can promote the regeneration of hippocampal neurons in epileptic rats and has neuroprotective effect [23]. Therefore, we chose the sensitive part of hippocampus as the research site to observe the hippocampal injury in each group. These aforementioned characteristics makes BDNF being a significant factor in reward-related processes, learning and memory, circuit formation as well as cognitive function. It is reported that both miRNAs and BDNF exert functions in neuronal processes, thus the crosstalk between both of which is very interesting [24]. Based on which, this study is performed to explore the effects of miR-103a on astrocytes activation and hippocampal neuron injury in epilepsy rats by binding to BDNF.

## Materials and methods

### Ethical statement

The experiment was approved by the animal ethics committee of Shanghai Pudong New Area People’s Hospital.

### Experimental animals and grouping

A total of 90 clean-grade and healthy Sprague–Dawley (SD) rats which weighted (230 ± 20) g were purchased from Beijing Vital River Laboratory Animal Technology Co., Ltd. (Beijing, China). After adaptive feeding for 1 week, the rats were kept in clean-grade animal room with the temperature of (22–24) °C, normal circadian rhythm, as well as free access to eating and drinking. According to the body weight, the rats were randomly assigned into six groups, with 15 rats in each group, namely the sham group (intraperitoneal injection of normal saline), the EP group (epilepsy rats without other treatment), the inhibitors negative control (NC) group (intracerebroventricular injection of inhibitors NC 24 h before EP modeling), miR-103a inhibitors + siRNA-NC group (intracerebroventricular injection of miR-103a inhibitors and siRNA-NC 24 h before EP modeling) and miR-103a inhibitors + BDNF-siRNA group (intracerebroventricular injection of miR-103a inhibitors and BDNF-NC 24 h before EP modeling). miR-103a inhibitors, inhibitors NC, BDNF-siRNA and siRNA-NC plasmids were all purchased from Shanghai GenePharma Co., Ltd (Shanghai, China).

### Establishment of a rat model of epilepsy

The rats were intraperitoneally injected with 127 mg/kg lithium chloride (LiCl), and 16–18 h later, intraperitoneally injected with 30 mg/kg pilocarpine (PILO). At 30 min before administration of PILO, the rats were intraperitoneally injected with 1 mg/kg atropine. According to the Racine classification, successful modeling was achieved if the attack progression reached grade IV or above, and the duration of which should sustain for 30 min or more. Those who did not succeed were added once per 30 min according to the original dose, and those who did not succeed with additional three times are excluded. At 60 min after the successful modeling, the rats were intraperitoneally injected with 10 mg/kg diazepam for spasmolysis. If the spasmolytic effect was not good, it should be added once per 60 min according to the original dose. After successful spasmolysis, the rats were intracerebroventricularly injected with miR-103a inhibitors, inhibitors NC, BDNF-siRNA and siRNA-NC, while the rats in the sham group had intraperitoneal injection of the same amount of normal saline.

### Sample collection

After 24 h of experimental treatment, the rats were intraperitoneally injected with 1% pentobarbital sodium for anesthesia. The jugular vein blood was extracted from five rats in each group, which was centrifuged for 10 min at 1500 r/min after resting for 30 min. Besides, the supernatant was stored at − 70 °C for further use. The levels of inflammatory factors (IL-6 and TNF-α) in serum were determined by enzyme-linked immunosorbent assay (ELISA) (Nanjing Jiancheng Bioengineering Institute, Nanjing, Jiangsu, China). Afterwards, the rats were kept at supine fixation with the chest opened to expose the heart. The needle was inserted into the ascending aorta through the left ventricle and the right atrial appendage was cut open. Subsequently, 150 mL cooled phosphate buffer saline (PBS) was infused within 2 min to remove blood, and brain was removed quickly by craniotomy. After that, the hippocampus was quickly separated on the ice and then placed in a pre-labeled cryopreservation tube. The hippocampus was rapidly put into liquid nitrogen, and finally transferred to − 80 °C refrigerator for the detection of reverse transcription quantitative polymerase chain reaction (RT-qPCR) and western blot analysis. Meanwhile, the hippocampal tissues from another five rats in each group was fixed in 4% paraformaldehyde for 24 h, dehydrated, embedded, and sliced into 4 μm sections, which were used for histological detection. Furthermore, the hippocampal tissues of the remaining five rats in each group were isolated and observed by a transmission electron microscope.

### Immunohistochemical staining

No less than three slices of hippocampal tissues from each rat of each group were selected, rinsed with PBS for three times, each time for 10 min, fixed with 4% 1-phosphofructaldolase (PFA) for 10 min, rinsed with PBS for three times, each time for 5 min, blocked with 5% goat serum for 30 min, added with primary antibody of glial fibrillary acidic protein (GFAP) (Abcam, Cambridge, MA, USA), and incubated at 37 °C for 2 h. Subsequently, the slices of hippocampal tissues were added with the secondary antibody at 37 °C for 2 h, rinsed with PBS for three times, each time for 10 min, added with S-P compound, and incubated at 37 °C for 30 min. After that, the slices of hippocampal tissues were rinsed with PBS for three times, each time for 10 min, added with 0.05% diaminobenzidine (DAB) for coloration for 5 min, dehydrated, cleared and mounted. Under the same exposure condition, Image-Pro Plus 6.0 software was used to analyze the average number of positive cells in GFAP by randomly selecting five visual fields under 100 times of a light microscope.

### Hematoxylin–eosin (HE) staining

No less than three slices of hippocampal tissues from each rat of each group were dewaxed and hydrated. After HE staining, the histopathological examination was performed and photos were taken so as to observe the histopathological condition in hippocampal tissues of rats in each group.

### Nissl’s staining

No less than three slices of hippocampal tissues from each rat of each group were dewaxed to distilled water. The slices were stained with preheated 1% toluidine blue (60 °C) for 40 min, rinsed with distilled water for 3 min, and then decolorized in gradient alcohol for 3 min each time to see if the Nissl granules were clear under the microscope, and the background which showed light blue to colorless was considered to be appropriate. Subsequently, the slices were dehydrated with gradient ethanol, cleared by xylene and sealed by neutral balsam after drying. The Nissl bodies in nerve cells were stained bluish violet under a light microscope. Image-Pro Plus was used to analyze the pictures, and the nerve cell count in hippocampal CA1 area was counted.

### Terminal deoxynucleotidyl transferase (TdT)-mediated dUTP nick end labeling (TUNEL) staining

No less than three slices of hippocampal tissues from each rat of each group were dehydrated with gradient ethanol, rinsed with PBS for three times, each time for 3 min. The slices were immersed into 200 mL 0.1 mol/L sodium citrate and heated in a 350 W microwave for 5 min; and it was taken out immediately and poured into 80 mL distilled water. After natural cooling, the slices were rinsed with PBS for three times, each time for 3 min. After the slide was dried, the samples were added with the TUNEL reaction mixture, and the sealing membrane was supplemented to react for 30 min at a 37 °C dark humidity box, and rinsed with PBS for three times, each time for 3 min. Afterwards, the samples were added with converter-peroxidase (POD), and the sealing membrane was supplemented to react for 30 min at a 37 °C dark humidity box, and rinsed with PBS for three times, each time for 3 min. Next, the samples were added with DAB substrate, and then placed at room temperature for 15 min, followed by washing with PBS for three times, each time for 3 min. The samples were then experienced hematoxylin counterstaining, returning blue by flowing water, dehydration by gradient ethanol, clearing by xylene and sealing by neutral balsam. The number of positive cells in CA1 region was counted when there were brown granules in the nucleus.

### Transmission electron microscope observation

With the application of glutaraldehyde-osmium tetroxide fixation, the rat hippocampal tissues were fixed with 2.5% glutaraldehyde and then added with 1% osmic acid for fixation. Afterwards, the tissues were dehydrated with acetone gradient, embedded with Epon 812 epoxy resin, and sliced into ultra-thin sections by a ultra-thin slicer, which was followed by the staining of lead citrate. The hippocampal neurons were observed under a JEM-1400Plus electron microscope.

### RT-qPCR

The one-step method of Trizol (Invitrogen, Carlsbad, CA, USA) was used to extract the total RNA of the hippocampus, and the high-quality RNA was confirmed by ultraviolet (UV) analysis and formaldehyde denaturation electrophoresis detection. After the acquirement of l μg RNA, cDNA was obtained by avian myeloblastosis virus reverse transcriptase (AMV-RT). PCR primer was designed and synthesized by Invitrogen, Carlsbad, CA, USA (Table 1). U6 and β-actin were used as internal controls. The PCR amplification conditions were as follows: pre-denaturation at 94 °C for 5 min, a total of 40 cycles of denaturation at 94 °C for 40 s, annealing at 60 °C for 1 min, extension at 72 °C for 1 min and finally, extension at 72 °C for 10 min. The product was confirmed by agarose gel electrophoresis. The threshold cycle (Ct) value of each reaction tube was obtained by manually selecting the threshold at the lowest point of parallel rise of each logarithmic expansion curve. 2^−ΔΔCt^ method was used to analyze the ratio relation of target gene expression between the experimental group and the control group. The formula is as follows: ΔΔCt = [Ct_(target gene)_ − Ct_(internal control gene)_]_the experimental group_ − [Ct_(target gene)_ − Ct_(internal control gene)_]_the control group_. The experiment was repeated for three times to obtain the average value.

**Table 1:** Primer sequence

Gene	Sequence
miR-103a	F: 5′-ACACTCCAGCTGGGAGCAGCATTGTACAGGGC-3′
	R: 5′-TGGTGTCGTGGAGTCG-3′
U6	F: 5′-CGCTTCGGCAGCACATATAC-3′
	R: 5′-AAATATGGAACGCT-TCACGA-3′
BDNF	F: 5′-GCTGCTGGATGAGGACCAGA-3′
	R: 5′-GCTGCTGGATGAGGACCAGA-3′
GFAP	F: 5′-GGAGTGGTATCGGTCTAAGTTTGC-3′
	R: 5′-GTTGGCGGCGATAGTCGTTAG-3′
IL-6	F: 5′-AGCCCACCAGGAACGAAAG-3′
	R: 5′-GGAAGGCAGTGGCTGTCAA-3′
TNF-α	F: 5′-CTGTGAAGGGAATGGGTGTT-3′
	R: 5′-GGGCTGGCTCTGTGAGGAAG-3′
β-Actin	F: 5′-GCCTTCCTTCTTGGGTAT-3′
	R: 5′-GGCATAGAGGTCTTTACGG-3′

miR-103a, microRNA-103a; BDNF, brain-derived neurotrophic factor; GFAP, glial fibrillary acidic protein

### Western blot analysis

The proteins from the hippocampal tissues were extracted and the protein concentrations were determined according to the instructions of the bicinchoninic acid (BCA) assay (Wuhan Boster Biological Technology LT, Wuhan, China). The extracted protein was added to the sample buffer and then boiled at 95 °C for 10 min, with each well for 30 μg protein. Following 10% sodium dodecyl sulfate polyacrylamide gel electrophoresis (SDS-PAGE) (Wuhan Boster Biological Technology LT, Wuhan, China), protein samples were transferred to a nitrocellulose membrane using the wet transfer method, with the electrophoretic voltage from 80 to 120 V, the trarsmembrane voltage of 100 mv and the time for 45–70 min. Subsequently, the protein samples were transferred to polyvinylidene fluoride (PVDF) membrane and blocked with 5% bovine serum albumin (BSA). Afterwards, the membranes were added with the primary antibodies of BDNF, GFAP and β-actin (1:3000; Abcam, Cambridge, MA, USA) and incubated at 4 °C overnight. The membranes were rinsed with Tris-buffered saline and Tween 20 (TBST) for three times, each time for 5 min, and the corresponding secondary antibodies (Shanghai Miao Tong Biotechnology Company, Shanghai, China) were incubated at room temperature for 1 h to wash the membranes for three times, each time for 5 min. An electrogenerated chemiluminescence (ECL) solution was used for developing. β-Actin was regarded as an internal control. Bio-rad Gel Dol EZ formatter (GEL DOC EZ IMAGER, Bio-rad, California, USA) was used for developing. The gray value analysis of target band was analyzed by Image J software (National Institutes of Health, Bethesda, Maryland, USA). The experiment was repeated for three time to obtain the average value.

### Double luciferase report gene assay

Bioinformatics software (http://www.targetscan.org) was used to predict the targeting relationship between miR-103a and BDNF and the binding sites between miR-103a and BDNF 3′UTR. The sequence of BDNF 3′UTR promoter containing miR-103a binding site was synthesized, and the BDNF 3′UTR wild-type (WT) plasmid was constructed. On the basis of this plasmid, the BDNF 3′UTR mutant (MUT) plasmid was constructed at the mutation binding site. According to the methods of the plasmid extraction kit (Promega, Madison, Wisconsin USA), the cells in the logarithmic growth were inoculated into 96-well plates and transfected with Lipofectamine 2000 at about 70% cell confluence. BDNF-WT and BDNF-MUT were mixed with mimics NC and miR-103a mimics (Shanghai GenePharma Co., Ltd (Shanghai, China)) respectively, and then co-transfected into 293T cells. After 48 h of transfection, the cells were collected and lysed. The luciferase activity was detected by luciferase detection kit (BioVision, San Francisco, CA, USA) and Glomax 20/20 luminometer (Promega, Madison, Wisconsin USA). The experiment was repeated three times.

### Statistical analysis

All the data were analyzed by SPSS 21.0 (IBM-SPSS, Inc, Chicago, IL, USA) software. The test of Kolmogorov–Smirnov verified that the data had a normal distribution. The results were expressed as mean ± standard deviation. The t test was used for the comparison between the two groups, and one-way analysis of variance (ANOVA) was used for the comparison among multiple groups. After ANOVA analysis, the Fisher’s least significant difference t test (LSD-t) was used for pairwise comparison. All tests were two-sided and *P* values ≤ 0.05 were considered statistically significant.

## Results

### Overexpression of miR-103a is found in hippocampus tissues of epilepsy rats

**Fig. 1:**
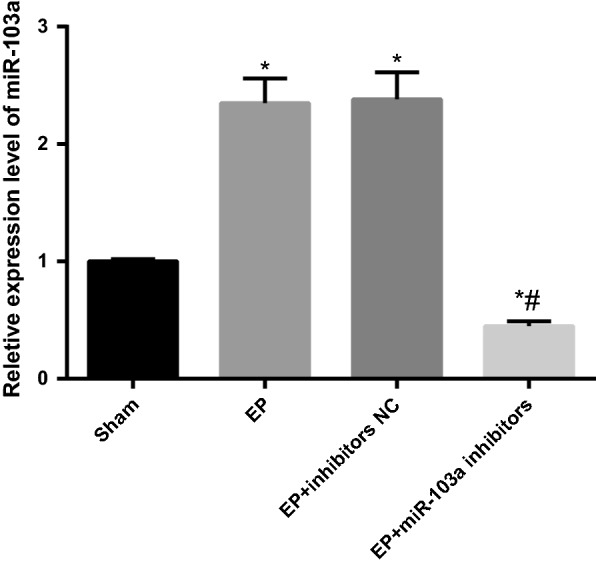
Expression of miR-103a in hippocampus tissues of rats in each group. N = 5, one-way analysis of variance (ANOVA) was used for the comparison among multiple groups. After ANOVA analysis, the Fisher’s least significant difference t test (LSD-t) was used for pairwise comparison. *P < 0.05 vs. the sham group; ^#^*P* < 0.05 vs. the EP + inhibitors NC group

RT-qPCR was used to detect the expression of miR-103a in hippocampus tissues of rats in each group. The results indicated that compared with the sham group, the expression of miR-103a increased significantly in hippocampus tissues of rats in the EP group (*P* < 0.05). Compared with the EP group, there was no significant difference in the expression of miR-103a in the hippocampus tissues of the EP + inhibitors NC group (*P *> 0.05). Compared with the EP + inhibitors NC group, the expression of miR-103a in the hippocampus tissues of the EP + miR-103a inhibitors was significantly reduced (*P* < 0.05; Fig. 1). The results showed that miR-103a was related to the development of epilepsy rats, and miR-103a inhibitors effectively interfered with the expression of miR-103a in hippocampus tissues of epilepsy rats.

### Downregulation of miR-103a inhibits activation of astrocytes in hippocampus tissues of epilepsy rats

**Fig. 2:**
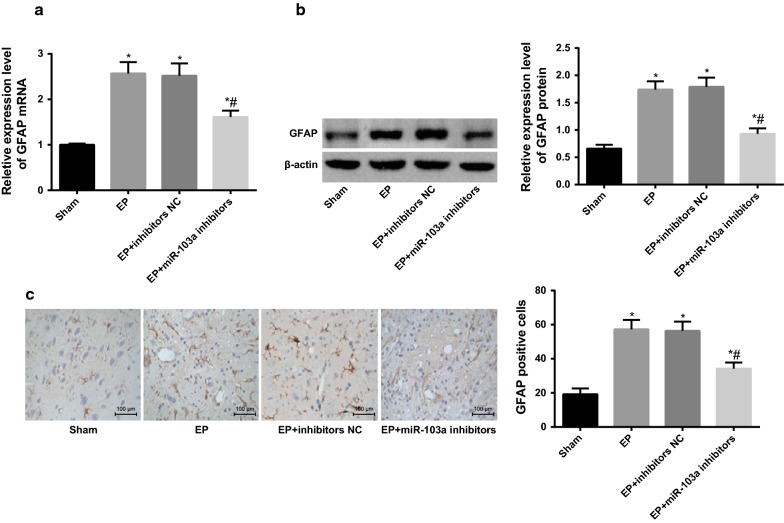
Expression of GFAP in hippocampus tissues of rats in each group. **a** The mRNA expression of GFAP in hippocampus tissues of rats was detected by RT-qPCR; **b** western blot analysis was used to detect the protein expression of GFAP in the hippocampus tissues of rats; **c** immunohistochemistry was used to detect the positive expression of GFAP in the hippocampus tissues of rats (×100). *P < 0.05 vs. the sham group; ^#^*P* < 0.05 vs. the EP + inhibitors NC group. N = 5, one-way analysis of variance (ANOVA) was used for the comparison among multiple groups. After ANOVA analysis, the Fisher’s least significant difference t test (LSD-t) was used for pairwise comparison

RT-qPCR and western blot analysis were used to detect the mRNA and protein expression of the astrocyte activation marker GFAP in the hippocampus tissues of rats in each group. It was found that the mRNA and protein expression of GFAP in the EP group was significantly increased compared with the sham group (*P* < 0.05). There was no significant difference in the mRNA and protein expression of GFAP between the EP group and the EP + inhibitors group (*P* > 0.05). The mRNA and protein expression of GFAP in the EP + miR-103a inhibitors group were significantly lower than those in the EP + inhibitors NC group (*P* < 0.05; Fig. 2a, b).

The activation of astrocytes in the hippocampus tissues of rats in each group was detected by immunohistochemistry. The results showed that the number of GFAP positive cells in the hippocampus tissues of the EP group was significantly larger than that of the sham group (*P* < 0.05), and there was no statistical difference in the number of GFAP positive cells in the hippocampus tissues of the EP group and the EP + inhibitors NC group (*P* > 0.05). The number of GFAP positive cells in the hippocampus tissues of the EP + miR-103a inhibitors group was significantly less than that in the EP + inhibitors NC group (*P* < 0.05; Fig. 2c), indicating that the inhibition of the expression of miR-103a could inhibit the activation of astrocytes in the hippocampus tissues of epilepsy rats.

### Downregulation of miR-103a inhibits the pathological injury of hippocampal neurons in epilepsy rats

**Fig. 3:**
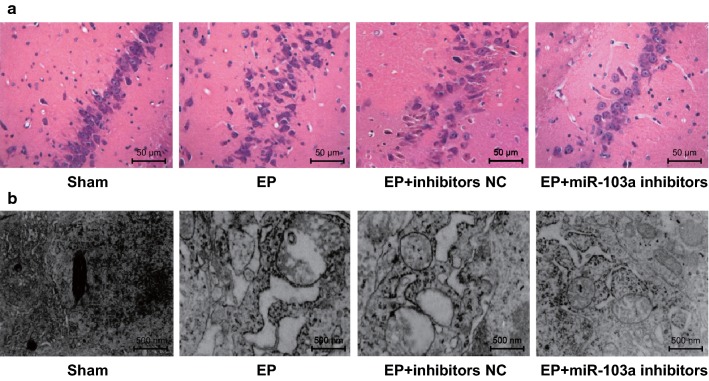
The pathological and ultrastructural changes in hippocampus tissues of rats in each group. N = 5; **a** pathological changes of hippocampal tissues of rats in each group (×200); **b** ultrastructure of hippocampal tissues of rats in each group (×20,000)

The results of HE staining in hippocampal tissue sections showed that: in the sham group, there were more neurons in the hippocampus tissues, and the morphology of cells was regular and orderly; in the EP and EP + inhibitors NC groups, the number of pyramidal cells in CA3 region, granulosa cells in DG area and small cone cells in CA1 area decreased, the cell morphology was irregular, the gap was obviously increased, the permutation was disorderly, the cytoplasm staining was deepened, and the nucleus was condensed and broken; in the EP + miR-103a inhibitors group, the neurons in the hippocampus tissues were swelling to some extent, the number of neurons decreased slightly, and the degree of pathological damage was significantly less than that of the EP + inhibitors NC group (Fig. 3a).

The ultrastructure of hippocampal tissues in rats was observed by a transmission electron microscope: the ultrastructure of hippocampal neurons in the sham group was normal, the structure of the cells was complete, the nucleolus was in the middle, and the chromatin was distributed evenly; the hippocampal neurons in the EP and EP + inhibitors NC groups appeared to have chromatin set, dissolution, mitochondria swelling, vacuolation and endoplasmic reticulum dilation; there were slight mitochondrial swelling and vacuolation in the EP + miR-103a inhibitors group (Fig. 3b), which suggested that inhibiting the expression of miR-103a could reduce the damage of hippocampal neurons in epilepsy rats.

### Downregulation of miR-103a promotes the survival of hippocampal neurons and inhibits its apoptosis in epilepsy rats

**Fig. 4:**
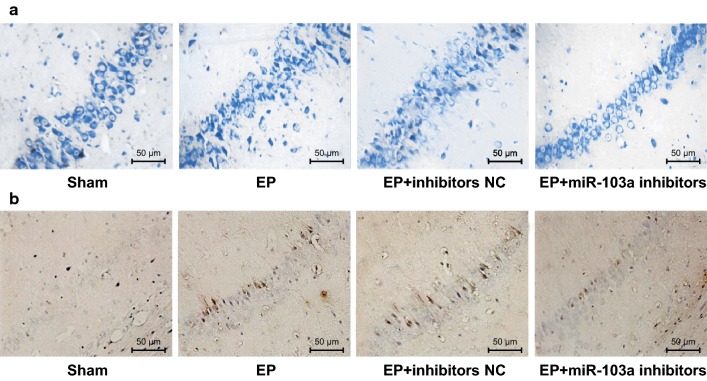
Survival and apoptosis of hippocampal neurons in rats of each group. **a** The survival of hippocampal neuronal in rats was detected by Nissl staining (×200); **b** The apoptosis of hippocampal neurons in rats was detected by TUNEL staining (×200). *P < 0.05 vs. the sham group; ^#^*P* < 0.05 vs. the EP + inhibitors NC group. N = 5, one-way analysis of variance (ANOVA) was used for the comparison among multiple groups. After ANOVA analysis, the Fisher’s least significant difference t test (LSD-t) was used for pairwise comparison

The survival of hippocampal neurons in each group was detected by Nissl staining. Compared with the sham group, the survival neurons decreased significantly in the hippocampus tissues of the EP and EP + inhibitors NC groups (both *P* < 0.05), while the survival neurons in the hippocampus tissues of the EP + miR-103a inhibitors group increased significantly compared with the EP + inhibitors NC group (*P* < 0.05; Fig. 4a).

The results of TUNEL staining revealed that there was fewer apoptotic neurons in the hippocampus tissues of the sham group. Compared with the sham group, the apoptosis of hippocampal neurons increased significantly in the EP and EP + inhibitors NC groups (both *P *< 0.05), but compared with the EP + inhibitors NC group, the apoptosis of hippocampal neurons decreased significantly in the EP + miR-103a inhibitors group (*P* < 0.05; Fig. 4b).

### Downregulation of miR-103a inhibits inflammatory injury in epilepsy rats

**Fig. 5:**
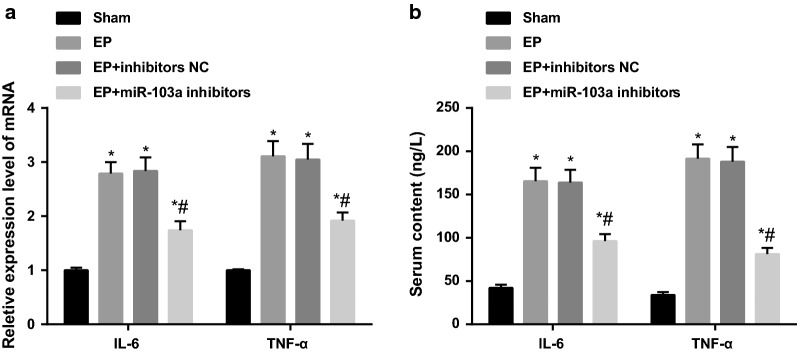
The levels of inflammatory factors in hippocampus tissues and serum of rats in each group. **a** RT-qPCR was used to detect the mRNA expression of inflammatory factors in the hippocampus tissues of rats; **b** the content of inflammatory factors in the serum of rats was detected by ELISA. *P < 0.05 vs. the sham group; ^#^*P* < 0.05 vs. the EP + inhibitors NC group. N = 5, one-way analysis of variance (ANOVA) was used for the comparison among multiple groups. After ANOVA analysis, the Fisher’s least significant difference t test (LSD-t) was used for pairwise comparison

The mRNA expression of inflammatory factors IL-6 and TNF-α in hippocampus tissues of each group was detected by RT-qPCR. The results showed that the mRNA expression of IL-6 and TNF-α in hippocampus tissues of the EP group was significantly increased compared with the sham group (both *P* < 0.05), and there was no statistical difference in the mRNA expression of IL-6 and TNF-α in the hippocampus tissues between the EP group and the EP + inhibitors NC group (both *P* > 0.05). Compared with the EP + inhibitors NC group, the mRNA expression of IL-6 and TNF-α decreased significantly in the hippocampus tissues of the EP + miR-103a inhibitors group (*P* < 0.05; Fig. 5a).

The contents of IL-6 and TNF-α in serum of rats in each group was detected by ELISA. The results suggested that there was no significant difference in the contents of IL-6 and TNF-α in serum of the EP and EP + inhibitors NC group (*P* > 0.05), but the contents of IL-6 and TNF-α in serum of the EP and EP + inhibitors NC groups was significantly higher than that in the sham group (both *P* < 0.05). The contents of IL-6 and TNF-α in serum of EP + miR-103a inhibitors group was significantly lower than that in the EP + inhibitors NC group (*P* < 0.05; Fig. 5b), indicating that the interference of miR-103a expression could reduce the inflammatory damage in epilepsy rats.

### Downregulation of miR-103a promotes the expression of BDNF in hippocampus tissues of epilepsy rats

**Fig. 6:**
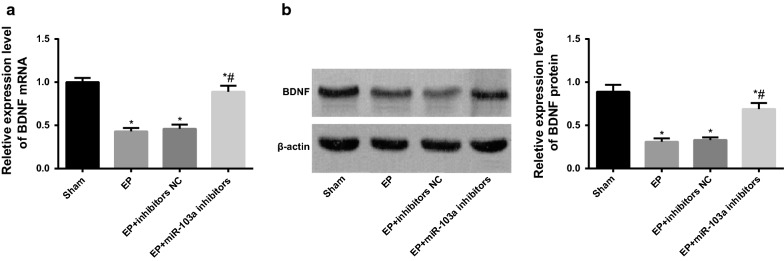
Expression of BDNF in hippocampal tissues of rats in each group. **a** RT-qPCR was used to detect the mRNA expression of BDNF in the hippocampus tissues of rats; **b** western blot analysis was used to detect the protein expression of BDNF in the hippocampus tissues of rats. *P < 0.05 vs. the sham group; ^#^*P* < 0.05 vs. the EP + inhibitors NC group. N = 5, one-way analysis of variance (ANOVA) was used for the comparison among multiple groups. After ANOVA analysis, the Fisher’s least significant difference t test (LSD-t) was used for pairwise comparison

RT-qPCR and western blot analysis were used to detect the mRNA and protein expression of BDNF in the hippocampus tissues of each group. The results showed that the mRNA and protein expressions of BDNF in the EP group and the EP + inhibitors NC group were significantly reduced in contrast to the sham group (both *P* < 0.05). The mRNA and protein expression of BDNF in the hippocampus tissues of rats increased significantly in the EP + miR-103a inhibitors group in comparison to the EP + inhibitors NC group (*P* < 0.05; Fig. 6), indicating that the inhibition of miR-103a could promote the expression of BDNF in the hippocampus tissues of epilepsy rats, and preliminarily suggested that miR-103a was associated with BDNF.

### Interfering BDNF reverses the activation of astrocytes in epilepsy rats by downregulating miR-103a expression

**Fig. 7:**
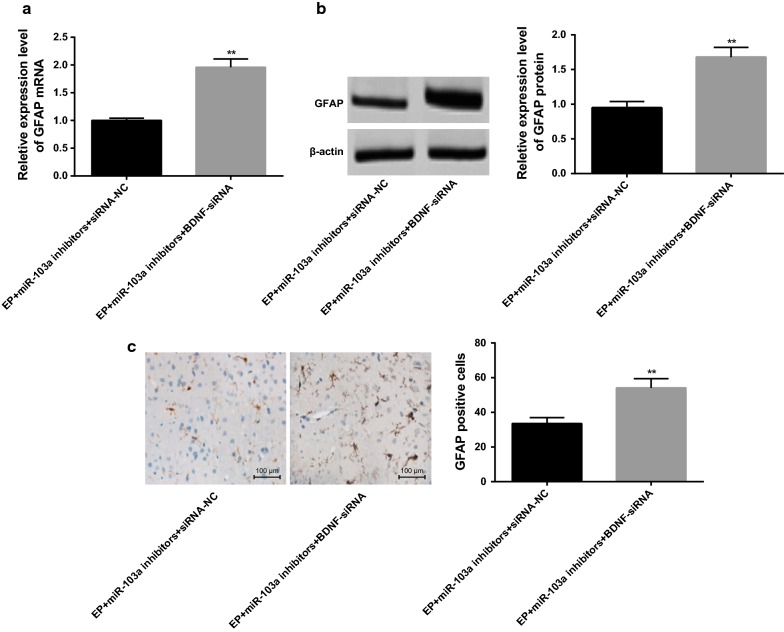
Expression of GFAP in hippocampus tissues of rats in two groups. **a** The mRNA expression of GFAP in the hippocampus tissues of rats in two groups was detected by RT-qPCR; **b** western blot analysis was used to detect the protein expression of GFAP in the hippocampus tissues of rats; **c** immunohistochemistry was used to detect the positive expression of GFAP in the hippocampus tissues of rats (×100); **P < 0.05 vs. the EP + miR-103a inhibitors + siRNA-NC group. N = 5, t test was used for data analysis

According to the results of RT-qPCR and western blot analysis, it suggested that compared with the EP + miR-103a inhibitors + siRNA-NC group, the expression of GFAP mRNA and protein in hippocampus tissues of rats was significantly increased in the EP + miR-103a inhibitors + BDNF-siRNA group (*P* < 0.01; Fig. 7a, b).

The results of immunohistochemistry showed that the number of GFAP positive cells in hippocampal tissues of rats in the EP + miR-103a inhibitors + BDNF-siRNA group was significantly higher than that in the EP + miR-103a inhibitors + siRNA-NC group (*P *< 0.01) (Fig. 7c), which indicates that inhibiting the expression of BDNF could block the effect of miR-103a inhibitors on the activation of astrocytes in hippocampus tissues of epilepsy rats.

### Interfering BDNF reverses the hippocampal neuron injury in epilepsy rats by downregulating miR-103a expression

**Fig. 8:**
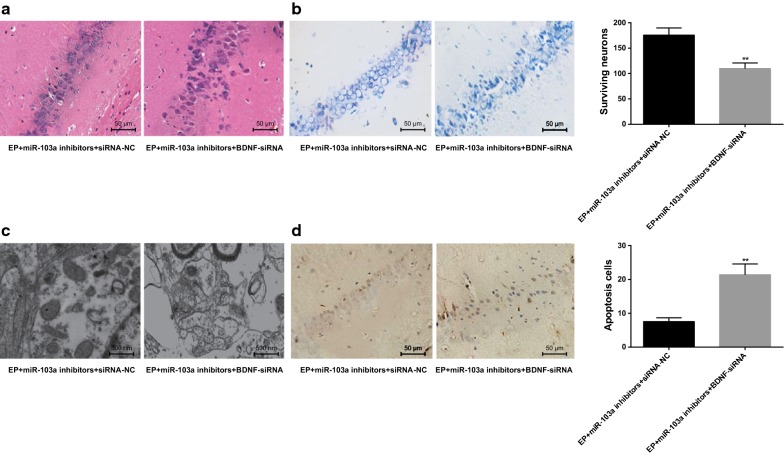
Changes of hippocampal neurons in rats of two groups. **a** HE staining was used to observe the histopathology of hippocampal tissues in two groups (×400); **b** ultrastructure of hippocampal tissues in two groups observed by a transmission electron microscope (×20,000); **c** the number of surviving neurons in the hippocampus tissues of the two groups was detected by Nissl staining (×200); **d** TUNEL staining was used to detect the number of neuronal apoptosis in hippocampal tissues of the two groups (×400); **P < 0.05 vs. the EP + miR-103a inhibitors + siRNA-NC group. N = 5, t test was used for data analysis

Results of HE staining indicated that compared with the EP + miR-103a inhibitors + siRNA-NC group, the pathological changes of hippocampal tissues in rats were obviously aggravated, and the number of nerve cells decreased significantly in the EP + miR-103a inhibitors + BDNF-siRNA group (Fig. 8a).

The results of transmission electron microscopy observation showed that the mitochondria swelling, vacuolation and endoplasmic reticulum dilatation in hippocampal tissues of rats in the EP + miR-103a inhibitors + BDNF-siRNA group were more serious than those in the EP + miR-103a inhibitors + siRNA-NC group (Fig. 8b).

The results of Nissl staining indicated that compared with the EP + miR-103a inhibitors + siRNA-NC group, the number of surviving neurons in hippocampus tissues of rats was significantly decreased in the EP + miR-103a inhibitors + BDNF-siRNA group (*P* < 0.01) (Fig. 8c).

The results of TUNEL staining showed that the number of apoptotic neurons in hippocampal tissues in the EP + miR-103a inhibitors + BDNF-siRNA group was significantly higher than that in the EP + miR-103a inhibitors + siRNA-NC group (*P* < 0.01) (Fig. 8d). These results suggest that interfering with BDNF can reverse the protective effect of down-regulation of miR-103a on hippocampal neuron injury in epilepsy rats.

### Interfering BDNF reverses the inhibitory effect of inflammatory injury in epilepsy rats by downregulating miR-103a expression

**Fig. 9:**
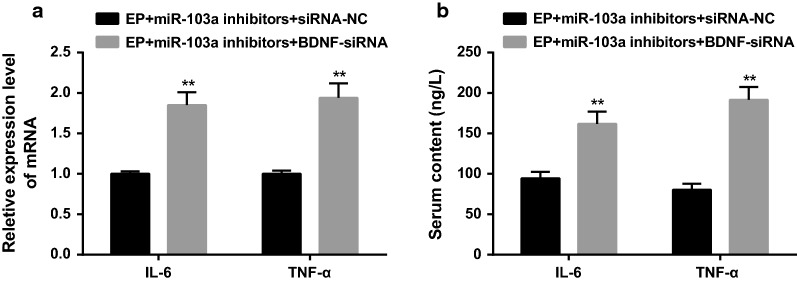
Levels of inflammatory factors in hippocampus tissues and serum of rats in two groups. **a** Detection of inflammatory factors in hippocampus tissues of two groups by RT-qPCR; **b** detection of the protein contents of inflammatory factors in serum by ELISA; **P < 0.05 vs. the EP + miR-103a inhibitors + siRNA-NC group. N = 5, t test was used for data analysis

According to the results of RT-qPCR and ELISA, it found that the mRNA expressions of IL-6 and TNF-α in hippocampus tissues and the protein contents of IL-6 and TNF-α in serum in the EP + miR-103a inhibitors + BDNF-siRNA group were significantly higher than those in the EP + miR-103a inhibitors + siRNA-NC group (all *P* < 0.01) (Fig. 9a, b).

### BDNF is a direct target gene of miR-103a

**Fig. 10:**
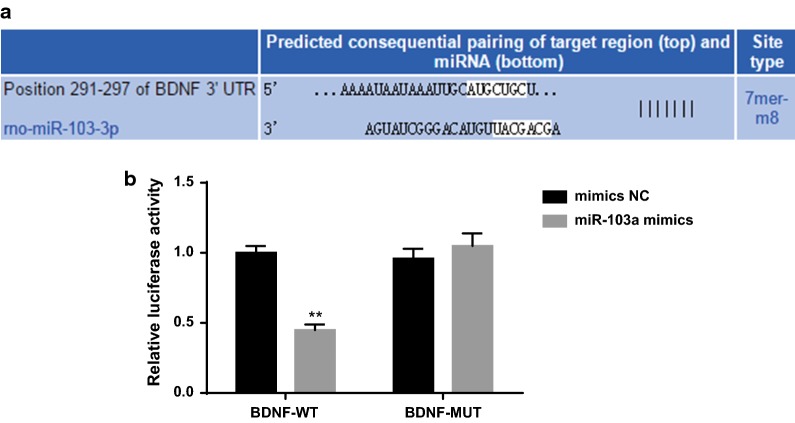
Target relationship between miR-103a and BDNF. **a** Online prediction software predicted the targeting relationship between miR-103a and BDNF; **b** experiment of luciferase activity to verify the targeting relationship between miR-103a and BDNF. The t test or the one-way analysis of variance (ANOVA) was used for comparison. After ANOVA analysis, the Fisher’s least significant difference t test (LSD-t) was used for pairwise comparison. Repetitions = 3; *P < 0.05 vs. the mimics NC group

The experimental results suggested that there may be a negative regulatory relationship between miR-103a and BDNF. A bioinformatics software http://www.targetscan.org was used to predict the bindings site between miR-103a and BDNF (Fig. 10a).

The results of luciferase activity determination showed that the relative luciferase activity of BDNF-3′UTR-WT + miR-103a mimics group was significantly lower than that of the BDNF-3′UTR-WT + mimics NC group (*P* < 0.05), while there was no significant difference in the relative luciferase activity between the BDNF-3′UTR-MUT + miR-103a mimics group and the BDNF-3′UTR-MUT + mimics NC group (*P* > 0.05) (Fig. 10b). The results indicate that BDNF is a direct target gene of miR-103a.

### Additional material

At the same time, we used RT-qPCR to detect the expression of miR-103, miR-107, miR-15, miR-16, miR-195, miR-497 of miR-15/107 family members in hippocampus of epileptic rats. The results showed that the expression of miR-103, miR-107, miR-15, miR-16, miR-195, and miR-497 in the hippocampus of epileptic rats was up-regulated compared with the sham group, and the expression of miR-103 was up-regulated (Additional file 1: Figure S1), so we selected miR-103a as the target miRNA of the present study.

## Discussion

Brain-specific miRNAs is important in synaptic function, and BDNF is a key part in synaptic plasticity in neurons, thus the interaction between brain-specific miRNAs and BDNF is a hot issue now [24]. Additionally, BDNF is able to induce upregulation of miR-132 in cultured cortical neurons, and upregulated miR-132 promotes the outgrowth of primary neurites, and there is a substantial decrease in neurite outgrowth with the transfection of an antisense RNA for miR-132 [25]. In our previous work, we found that the expression of miR-103, miR-107, miR-15, miR-16, miR-195, and miR-497 in the hippocampus of epileptic rats was up-regulated compared with the sham group, and the expression of miR-103 was up-regulated, so we selected miR-103a as the target miRNA of the present study. However, the specific mechanism of miR-103a in epilepsy remains to be discovered. Therefore, our study is supposed to investigate the role of miR-103a in epilepsy by regulating the expression of BDNF. The results demonstrated that inhibition of miR-103a can inhibit the activation of astrocytes in hippocampus tissues and improve the pathological injury of neurons of epilepsy rats through the targeted regulation of BDNF gene.

One of the most important findings in this study revealed that upregulated miR-103a and downregulated BDNF are found in hippocampus of epilepsy rats. The functions of one special miRNA, namely miR-103a, have been reported to be overexpressed in several cancer cell lines, such as endometrial cancer, pancreatic cancer and bladder cancer [26–28]. Several evidence has elucidated that miR-103 may act as a novel oncogene in human cancers [29, 30]. Meanwhile, BDNF is important for the survival of the neuronal populations during development, and it is involved in the axonal and dendritic growth and synaptogenesis [31]. It is suggested that decreased expression of BDNF are related to depression, and which will become enhanced after antidepressant treatment [32, 33]. Additionally, according to the results of online software and luciferase activity determination, we found that BDNF was a direct target gene of miR-103a. Similar to our study, miR-103 was determined to target a gene (PER3) in colorectal cancer cells, which induces apoptosis of cancer cells, and also, the tumor suppressor genes of DICER and PTEN [34, 35]. Except that, studies also demonstrated that silencing of miR-103 suppressed proliferation of mouse intestinal cell through targeting the CCNE1, CDK2, as well as CREB1 genes [36, 37]. Seven miRNAs in miR-103/107 family are involved in regulating the expression of BDNF in human prefrontal cortex (PFC) [38]. BDNF is closely related to many neurological diseases such as Alzheimer’s disease and depression. The level of BDNF mRNA in human PFC increased from early childhood to early adulthood, and remained at about the same level after adulthood, while the level of BDNF protein decreased gradually with the aging of human PFC [38]. At the same time, some experts reported that in the model of epilepsy induced by pilocarpine, the increase of BDNF concentration could inhibit the occurrence of epilepsy [39]. Recently, it has been reported that: compared with the control group, the seizure degree and the peak value of abnormal electroencephalogram (EEG) decreased in the rat model of epilepsy treated with BDNF, which indicated that the sustained low concentration of BDNF could relieve the seizure. At the same time, it was also found that BDNF could promote the regeneration of hippocampal neurons and had neuroprotective effect [23]. It can be inferred that miR-103, as a post-transcriptional inhibitor, can inhibit the expression of BDNF in the brain of epileptic rats, resulting in a decrease in the expression of BDNF in the hippocampus of epileptic rats, thus affecting the damage of hippocampal neurons.

Our study also demonstrated that inhibiting miR-103a expression can inhibit the activation of astrocytes in epilepsy rats, which is reflected by the results that the mRNA and protein expression of GFAP were significantly lower in the EP + miR-103a inhibitors group. GFAP, expresses almost exclusively in astrocytes, is an essential factor in malignancy progression of brain neoplasms, which serves as a vital component of cytoskeleton [40]. A growing number of evidences revealed that GFAP level increased in high-grade brain tumors, implying the important role of GFAP in the aggressiveness of brain tumors [41–43]. It is reported that via interfering the binding of miR-139 and 3′UTR, variant G allele of rs11558961 that reduced GFAP expression contributes to low GFAP expression, and further inhibits the chemo-resistance and metastasis of glioblastoma (GBM) cells [44]. Additionally, the results of our study suggested that inhibiting miR-103a expression can attenuate injury of hippocampal neurons in epilepsy rats, which is reflected by the results that the mRNA expression of IL-6 and TNF-α decreased significantly in the hippocampus tissues of rats in the EP + miR-103a inhibitors group. Evidence has shown that cytokines are involved in the pathogenesis of epilepsy [45]. A large number of clinical and basic studies have also confirmed that inflammatory factors and inflammatory reactions are involved in the pathogenesis of epilepsy [46, 47]. TNF-α is a cytokine with a wide range of biological functions, and it is a common medium of nerve and immune system and participates in immune response and inflammation. TNF-α is expressed at a high level during epilepsy, and the specific mechanism of its action in epilepsy may be related to glial cells [48]. At present, a study has shown that IL-6 has neuroprotective and neurotrophic effects in the central nervous system. According to the study of IL-6-deficient mice, IL-6 plays a role as a neuroprotective factor in epileptic brain injury [49]. Furthermore, IL-6 and TNF-α were also selected as representatives of inflammatory injury in epileptic rats [50]. Therefore, this study also detected the expression of inflammatory factors IL-6 and TNF-α in epileptic rats. As was reported, the mRNA expression of TNF-α, IL-6 and IL-1β was decreased in rats of the electroacupuncture group compared with the vascular dementia (VD) group. Simultaneously, the degree of neuronal injury in hippocampus was decreased, and its morphology was basically restored to normal [51]. Also, miR-26 is suggested to largely regulate inflammation and tumourigenicity via down-regulating IL-6 production [52]. Indeed, in primary human fibroblasts, ectopic expression of miR-146a/b suppressed IL-6 and IL-8 secretion as well as downregulated IRAK1, which is an essential factor of the IL-1 receptor signaling pathway [53]. Furthermore, our study elucidated that interfering BDNF reverses the activation of astrocytes and the hippocampal neuron injury in epilepsy rats by downregulating miR-103a expression.

## Conclusion

Collectively, this present study supports that inhibition of miR-103a can inhibit the activation of astrocytes in hippocampus tissues of epilepsy rats and improve the pathological injury of neurons, and the mechanism of which is related to targeted regulation of BDNF gene. However, our study also performed in animals, further study which concentrated on cell levels could further verify our result.

## Additional file

**Additional file 1: Figure S1.** Expression levels of miR-103, miR-107, miR-15, miR-16, miR-195, miR-497 in the hippocampus of epileptic rats. *P < 0.05 vs. the sham group.
