# Correction to: Inhibition of microRNA-103a inhibits the activation of astrocytes in hippocampus tissues and improves the pathological injury of neurons of epilepsy rats by regulating BDNF

**DOI:** 10.1186/s12935-021-02238-7

**Published:** 2021-10-26

**Authors:** Ping Zheng, He Bin, Wei Chen

**Affiliations:** 1 Department of Neurosurgery, Shanghai Pudong New Area People’s Hospital, No. 490, South Chuanhuan Road, Shanghai, 201299 People’s Republic of China; 2 Department of Neurosurgery, First Afliated Hospital of Nanchang University, Nanchang, China

## Correction to: Cancer Cell Int 19:109 (2019)10.1186/s12935-019-0821-2

Following the publication of the original article [1], we were notified of a misalignment in Fig. 10a.

The corrected Fig. 10 (Fig. 10) is presented in this erratum.

**Fig. 10:**
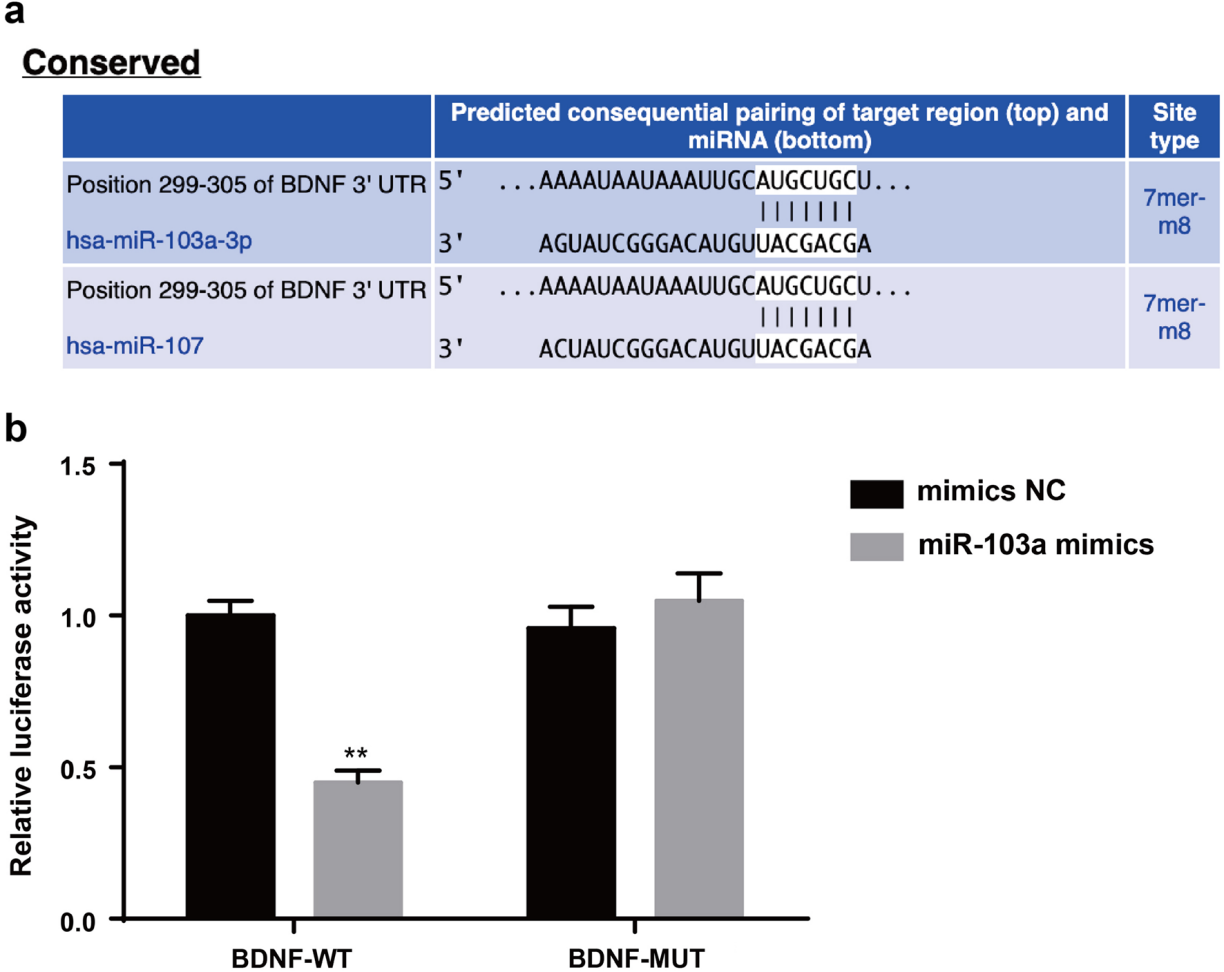
Target relationship between miR-103a and BDNF. **a** Online prediction software predicted the targeting relationship between miR-103a and BDNF. **b** Experiment of luciferase activity to verify the targeting relationship between miR-103a and BDNF. The t test or the one-way analysis of variance (ANOVA) was used for comparison. After ANOVA analysis, the Fisher’s least significant difference t test (LSD-t) was used for pairwise comparison. Repetitions = 3; *P  <  0.05 vs. the mimics NC group
